# Supplementary material for: Genetic characterization of the prion protein gene in camels (Camelus) with comments on the evolutionary history of prion disease in Cetartiodactyla
Source: PeerJ. 2024 Jun 27;12:e17552. doi: 10.7717/peerj.17552 (PMC11214740; doi:10.7717/peerj.17552)
Supplement: Supplemental Information 1 — Tables 1–3 list all individuals and their associated data that were used in this study. [file peerj-12-17552-s001.docx]

Table 1. List of *Camelus dromedarius* used for characterization of the exon 3 region of the prion protein gene from Legesse et al. (2018). Pertinent information, including individual’s TK number, breed, locality, sex, and age, was obtained from data deposited into the Natural Science Research Laboratory at the Museum of Texas Tech University. ^1^ARSE = Afar Regional State of Ethiopia. ^2^ESRS = Ethiopian Somali Regional State. ^3^ORSE = Oromia Regional State of Ethiopia.

| TK Number | NCBI GenBank Accession Number | ID used in Legesse et al. (2018) | Breed | Distribution | Sex | Age |
| --- | --- | --- | --- | --- | --- | --- |
| 194733 | OP414499 | AF232 | Afar | Zone-3, ^1^ARSE | Female | 14 |
| 194743 | OP414500 | AF242 | Afar | Zone-3, ARSE | Male | 1 |
| 194751 | OP414501 | AF250 | Afar | Zone-3, ARSE | Male | 1 |
| 194744 | OP414504 | AF243 | Afar | Zone-3, ARSE | Male | 1 |
| 194745 | OP414503 | AF244 | Afar | Zone-3, ARSE | Male | 1 |
| 194746 | OP414502 | AF245 | Afar | Zone-3, ARSE | Female | 12 |
| 194735 | OP414505 | AF234 | Afar | Zone-3, ARSE | Female | 11 |
| 194742 | OP414506 | AF241 | Afar | Zone-3, ARSE | Male | 1 |
| 194723 | OP414507 | AF 222 | Afar | Zone-3, ARSE | Female | 9 |
| 194740 | OP414509 | AF239 | Afar | Zone-3, ARSE | Female | 9 |
| 194741 | OP414508 | AF240 | Afar | Zone-3, ARSE | Male | 1 |
| 194738 | OP414510 | AF237 | Afar | Zone-3, ARSE | Female | 10 |
| 194739 | OP414498 | AF238 | Afar | Zone-3, ARSE | Male | 1 |
| 194603 | OP414511 | A 99 | Ayden | Jarar, Korahe, and Shebelle zones, ^2^ESRS | Male | 12 |
| 194604 | OP414513 | A 100 | Ayden | Jarar, Korahe, and Shebelle zones, ESRS | Female | 12 |
| 194605 | OP414512 | A 101 | Ayden | Jarar, Korahe, and Shebelle zones, ESRS | Female | 9 |
| 194619 | OP414515 | A 115 | Ayden | Jarar, Korahe, and Shebelle zones, ESRS | Male | 16 |
| 194620 | OP414514 | A 116 | Ayden | Jarar, Korahe, and Shebelle zones, ESRS | Male | 6 |
| 194667 | OP414516 | B 166 | Borena | Borana zone, ^3^ORSE | Female | 13 |
| 194668 | OP414517 | B 167 | Borena | Borana zone, ORSE | Female | 9 |
| 194670 | OP414518 | B 169 | Borena | Borana zone, ORSE | Male | 7 |
| 194578 | OP414519 | H 74 | Hoor | Jarar, Korahe, and Shebelle zones, ESRS | Female | 11 |
| 194582 | OP414520 | H 78 | Hoor | Jarar, Korahe, and Shebelle zones, ESRS | Female | 12 |
| 194598 | OP414521 | H 94 | Hoor | Jarar, Korahe, and Shebelle zones, ESRS | Male | 13 |
| 194592 | OP414522 | H 88 | Hoor | Jarar, Korahe, and Shebelle zones, ESRS | Female | 10 |
| 194591 | OP414523 | H 87 | Hoor | Jarar, Korahe, and Shebelle zones, ESRS | Male | 10 |
| 194587 | OP414524 | H 83 | Hoor | Jarar, Korahe, and Shebelle zones, ESRS | Male | 5 |
| 194572 | OP414525 | H 68 | Hoor | Jarar, Korahe, and Shebelle zones, ESRS | Male | 11 |
| 194614 | OP414526 | H 110 | Hoor | Jarar, Korahe, and Shebelle zones, ESRS | Male | 5 |
| 194588 | OP414527 | H 84 | Hoor | Jarar, Korahe, and Shebelle zones, ESRS | Female | 9 |
| 194565 | OP414528 | H 61 | Hoor | Jarar, Korahe, and Shebelle zones, ESRS | Female | 9 |
| 194581 | OP414529 | H 77 | Hoor | Jarar, Korahe, and Shebelle zones, ESRS | Female | 17 |
| 194537 | OP414530 | I33 | Issa | Sitti zone, ESRS | Female | 11 |
| 194563 | OP414531 | I59 | Issa | Sitti zone, ESRS | Female | 8 |
| 194561 | OP414532 | I57 | Issa | Sitti zone, ESRS | Female | 10 |
| 194503 | OP414533 | J3 | Jigjiga | Jigjiga zone, ESRS | Male | 13 |
| 194506 | OP414534 | J6 | Jigjiga | Jigjiga zone, ESRS | Female | 10 |
| 194511 | OP414535 | J11 | Jigjiga | Jigjiga zone, ESRS | Female | 9 |
| 194697 | OP414536 | K196 | Kerreyu | East Shoa, ORSE | Female | 11 |
| 194702 | OP414537 | K201 | Kerreyu | East Shoa, ORSE | Male | 1 |
| 194703 | OP414540 | K202 | Kerreyu | East Shoa, ORSE | Female | 6 |
| 194704 | OP414538 | K203 | Kerreyu | East Shoa, ORSE | Male | 1 |
| 194712 | OP414539 | K211 | Kerreyu | East Shoa, ORSE | Female | 10 |
| 194632 | OP414543 | L 131 | Liben | Liben zone, ESRS | Male | 13 |
| 194633 | OP414541 | L132 | Liben | Liben zone, ESRS | Female | 14 |
| 194638 | OP414542 | L137 | Liben | Liben zone, ESRS | Female | 13 |
| 194639 | OP414547 | L138 | Liben | Liben zone, ESRS | Female | 8 |
| 194640 | OP414544 | L139 | Liben | Liben zone, ESRS | Female | 15 |
| 194642 | OP414545 | L141 | Liben | Liben zone, ESRS | Female | 12 |
| 194643 | OP414546 | L142 | Liben | Liben zone, ESRS | Female | 14 |

Table 2. List of individual camels representing *Camelus* used for characterization, comparison, and phylogenetic analyses of the exon 3 region of the prion protein gene from NCBI GenBank. Locality was included when available.

| NCBI GenBank Accession Number | Species | Locality |
| --- | --- | --- |
| AF113940 | *Camelus dromedarius* | Nuremberg Zoo, Germany |
| JX412230 | *C. dromedarius* | Iran |
| MF685344 | *C. dromedarius* | Egpyt |
| MF990557 | *C. dromedarius* | Sahraoui, Algeria |
| MF990558 | *C. dromedarius* | Sahraoui, Algeria |
| MF990559 | *C. dromedarius* | Sahraoui, Algeria |
| MK655460 | *C. dromedarius* | Azawad, Hybrid, and Rguibi, Algeria |
| MK655461 | *C. dromedarius* | Targui, Algeria |
| XM031434200 | *C. dromedarius* | Eithental, Austria |
| XM031434201 | *C. dromedarius* | Eithental, Austria |
| XM031434202 | *C. dromedarius* | Eithental, Austria |
| Y09760 | *C. dromedarius* | UnitedKingdom |
| AY723284 | *C. bactrianus* | China |
| AY723285 | *C. bactrianus* | China |
| HQ204566 | *C. bactrianus* | China |
| HQ204567 | *C. bactrianus* | China |
| JF514091 | *C. bactrianus* | China |
| JF514092 | *C. bactrianus* | China |
| JF514093 | *C. bactrianus* | China |
| JF514094 | *C. bactrianus* | China |
| JF514095 | *C. bactrianus* | China |
| JF514096 | *C. bactrianus* | China |
| JF514097 | *C. bactrianus* | China |
| JF514098 | *C. bactrianus* | China |
| JF514099 | *C. bactrianus* | China |
| JF514100 | *C. bactrianus* | China |
| JF514101 | *C. bactrianus* | China |
| JF514102 | *C. bactrianus* | China |
| JF514103 | *C. bactrianus* | China |
| JF514104 | *C. bactrianus* | China |
| JF514105 | *C. bactrianus* | China |
| JF514106 | *C. bactrianus* | China |
| JF514107 | *C. bactrianus* | China |
| JF514108 | *C. bactrianus* | China |
| JF514109 | *C. bactrianus* | China |
| JF514110 | *C. bactrianus* | China |
| JF514111 | *C. bactrianus* | China |
| JF514112 | *C. bactrianus* | China |
| JF514113 | *C. bactrianus* | China |
| JF514114 | *C. bactrianus* | China |
| JF514115 | *C. bactrianus* | China |
| JF514116 | *C. bactrianus* | China |
| JF514117 | *C. bactrianus* | China |
| JF514118 | *C. bactrianus* | China |
| XM010972734 | *C. bactrianus* | Inner Mongolia Autonomous Region, China |
| XM032461888 | *C. ferus* | Mongolia |

Supplemental Table 3. List of individuals used for outgroup designation (Order Chiroptera) and representative of the Orders Artiodactyla, Cetacea, and Perrisodactyla used for characterization, comparison, and phylogenetic analyses of the exon 3 region of the prion protein gene from NCBI GenBank.

| NCBI GenBank  Accession Number | Species | Taxonomic Classification |
| --- | --- | --- |
| XM024559617 | *Desmodus_rotundus* | Order Chiroptera |
| XM004433783 | *Ceratotherium simum* | Order Perissodactyla, Family Rhinocerotidae |
| AY133052 | *Diceros bicornis* | Order Perissodactyla, Family Rhinocerotidae |
| EF127815 | *Equus asinus* | Order Perissodactyla, Family Equidae |
| FJ746569 | *Equus asinus* | Order Perissodactyla, Family Equidae |
| FJ746570 | *Equus asinus* | Order Perissodactyla, Family Equidae |
| FJ746571 | *Equus asinus* | Order Perissodactyla, Family Equidae |
| EF165077 | *Equus asinus* | Order Perissodactyla, Family Equidae |
| EF165078 | *Equus asinus* | Order Perissodactyla, Family Equidae |
| XM044748276 | *Equus asinus* | Order Perissodactyla, Family Equidae |
| EF165074 | *Equus burchellii* | Order Perissodactyla, Family Equidae |
| EF165069 | *Equus burchellii* | Order Perissodactyla, Family Equidae |
| AF117317 | *Equus caballus* | Order Perissodactyla, Family Equidae |
| AY133051 | *Equus caballus* | Order Perissodactyla, Family Equidae |
| EF165070 | *Equus caballus* | Order Perissodactyla, Family Equidae |
| EF165071 | *Equus caballus* | Order Perissodactyla, Family Equidae |
| EU887254 | *Equus caballus* | Order Perissodactyla, Family Equidae |
| EU887255 | *Equus caballus* | Order Perissodactyla, Family Equidae |
| EU887256 | *Equus caballus* | Order Perissodactyla, Family Equidae |
| EU887257 | *Equus caballus* | Order Perissodactyla, Family Equidae |
| EU887258 | *Equus caballus* | Order Perissodactyla, Family Equidae |
| EU887259 | *Equus caballus* | Order Perissodactyla, Family Equidae |
| EU887260 | *Equus caballus* | Order Perissodactyla, Family Equidae |
| NM001143798 | *Equus caballus* | Order Perissodactyla, Family Equidae |
| XM023625924 | *Equus caballus* | Order Perissodactyla, Family Equidae |
| EF165075 | *Equus przewalskii* | Order Perissodactyla, Family Equidae |
| EU887243 | *Equus przewalskii* | Order Perissodactyla, Family Equidae |
| EU887244 | *Equus przewalskii* | Order Perissodactyla, Family Equidae |
| EU887245 | *Equus przewalskii* | Order Perissodactyla, Family Equidae |
| EU887246 | *Equus przewalskii* | Order Perissodactyla, Family Equidae |
| EU887247 | *Equus przewalskii* | Order Perissodactyla, Family Equidae |
| EU887248 | *Equus przewalskii* | Order Perissodactyla, Family Equidae |
| EU887249 | *Equus przewalskii* | Order Perissodactyla, Family Equidae |
| EU887250 | *Equus przewalskii* | Order Perissodactyla, Family Equidae |
| EU887251 | *Equus przewalskii* | Order Perissodactyla, Family Equidae |
| EU887252 | *Equus przewalskii* | Order Perissodactyla, Family Equidae |
| EU887253 | *Equus przewalskii* | Order Perissodactyla, Family Equidae |
| XM046678791 | *Equus quagga* | Order Perissodactyla, Family Equidae |
| AF117329 | *Equus quagga* | Order Perissodactyla, Family Equidae |
| EF165073 | *Equus zebra* | Order Perissodactyla, Family Equidae |
| XM005672669 | *Sus scrofa* | Order Artiodactyla, Suborder Suiformes, Family Suidae |
| L07623 | *Sus scrofa* | Order Artiodactyla, Suborder Suiformes, Family Suidae |
| EF139171 | *Sus scrofa* | Order Artiodactyla, Suborder Suiformes, Family Suidae |
| FJ161946 | *Sus scrofa* | Order Artiodactyla, Suborder Suiformes, Family Suidae |
| XM047771728 | *Phacochoerus africanus* | Order Artiodactyla, Suborder Suiformes, Family Suidae |
| AB919084 | *Hexaprotodon liberiensis* | Order Artiodactyla, Suborder Suiformes, Family Suidae |
| AY133053 | *Hippopotamus amphibius* | Order Artiodactyla, Suborder Suiformes, Family Suidae |
| AB919083 | *Hippopotamus amphibius* | Order Artiodactyla, Suborder Suiformes, Family Suidae |
| AF113943 | *Lama glama* | Order Artiodactyla, Suborder Tylopoda, Family Camelidae |
| KT692714 | *Vicugna pacos* | Order Artiodactyla, Suborder Tylopoda, Family Camelidae |
| KT692715 | *Vicugna pacos* | Order Artiodactyla, Suborder Tylopoda, Family Camelidae |
| XM007191700 | *Balaenoptera acutorostrata* | Order Cetacea, Suborder Mysticeti, Family Balaenopteridae |
| AB919078 | *Balaenoptera acutorostrata* | Order Cetacea, Suborder Mysticeti, Family Balaenopteridae |
| XM036825358 | *Balaenoptera musculus* | Order Cetacea, Suborder Mysticeti, Family Balaenopteridae |
| DQ884474 | *Balaenoptera physalus* | Order Cetacea, Suborder Mysticeti, Family Balaenopteridae |
| DQ884475 | *Balaenoptera physalus* | Order Cetacea, Suborder Mysticeti, Family Balaenopteridae |
| AB919080 | *Balaenoptera physalus* | Order Cetacea, Suborder Mysticeti, Family Balaenopteridae |
| DQ884473 | *Delphinus delphis* | Order Cetacea, Suborder Odontoceti, Family Delphinidae |
| XM030872630 | *Globicephala melas* | Order Cetacea, Suborder Odontoceti, Family Delphinidae |
| XM030872625 | *Globicephala melas* | Order Cetacea, Suborder Odontoceti, Family Delphinidae |
| DQ884471 | *Grampus griseus* | Order Cetacea, Suborder Odontoceti, Family Delphinidae |
| XM027118244 | *Lagenorhynchus obliquidens* | Order Cetacea, Suborder Odontoceti, Family Delphinidae |
| XM004276438 | *Orcinus orca* | Order Cetacea, Suborder Odontoceti, Family Delphinidae |
| XM012535450 | *Orcinus orca* | Order Cetacea, Suborder Odontoceti, Family Delphinidae |
| AB919071 | *Stenella attenuata* | Order Cetacea, Suborder Odontoceti, Family Delphinidae |
| AB919073 | *Stenella longirostris* | Order Cetacea, Suborder Odontoceti, Family Delphinidae |
| AB919075 | *Tursiops aduncus* | Order Cetacea, Suborder Odontoceti, Family Delphinidae |
| AF117311 | *Tursiops truncatus* | Order Cetacea, Suborder Odontoceti, Family Delphinidae |
| DQ130069 | *Tursiops truncatus* | Order Cetacea, Suborder Odontoceti, Family Delphinidae |
| DQ130070 | *Tursiops truncatus* | Order Cetacea, Suborder Odontoceti, Family Delphinidae |
| AB919063 | *Kogia breviceps* | Order Cetacea, Suborder Odontoceti, Family Kogiidae |
| AB919064 | *Kogia sima* | Order Cetacea, Suborder Odontoceti, Family Kogiidae |
| XM007463977 | *Lipotes vexillifer* | Order Cetacea, Suborder Odontoceti, Family Lipotidae |
| AB919060 | *Delphinapterus leucas* | Order Cetacea, Suborder Odontoceti, Family Monodontidae |
| XM022556485 | *Delphinapterus leucas* | Order Cetacea, Suborder Odontoceti, Family Monodontidae |
| XM029221058 | *Monodon monoceros* | Order Cetacea, Suborder Odontoceti, Family Monodontidae |
| XM024764250 | *Neophocaena asiaeorientalis* | Order Cetacea, Suborder Odontoceti, Family Phocoenidae |
| AB919069 | *Phocoena phocoena* | Order Cetacea, Suborder Odontoceti, Family Phocoenidae |
| XM032605446 | *Phocoena sinus* | Order Cetacea, Suborder Odontoceti, Family Phocoenidae |
| AB919070 | *Physeter catodon* | Order Cetacea, Suborder Odontoceti, Family Physeteridae |
| AY133054 | *Physeter catodon* | Order Cetacea, Suborder Odontoceti, Family Physeteridae |
| XM007123285 | *Physeter catodon* | Order Cetacea, Suborder Odontoceti, Family Physeteridae |
| DQ884467 | *Ziphius cavirostris* | Order Cetacea, Suborder Odontoceti, Family Ziphiidae |
| AF090852 | *Antilocapra americana* | Order Artiodactyla, Suborder Ruminantia, Family Antilocapridae |
| AY723286 | *Moschus chrysogaster* | Order Artiodactyla, Suborder Ruminantia, Family Moschidae |
| AF113942 | *Giraffa camelopardalis* | Order Artiodactyla, Suborder Ruminantia, Family Giraffidae |
| MT996365 | *Axis axis* | Order Artiodactyla, Suborder Ruminantia, Family Cervidae, Tribe Cervini |
| AF113945 | *Cervus canadensis* | Order Artiodactyla, Suborder Ruminantia, Family Cervidae, Tribe Cervini |
| Y09761 | *Cervus elaphus* | Order Artiodactyla, Suborder Ruminantia, Family Cervidae, Tribe Cervini |
| AY679697 | *Cervus elaphus* | Order Artiodactyla, Suborder Ruminantia, Family Cervidae, Tribe Cervini |
| MK103027 | *Cervus elaphus* | Order Artiodactyla, Suborder Ruminantia, Family Cervidae, Tribe Cervini |
| EU082263 | *Cervus elaphus* | Order Artiodactyla, Suborder Ruminantia, Family Cervidae, Tribe Cervini |
| EU082287 | *Cervus elaphus* | Order Artiodactyla, Suborder Ruminantia, Family Cervidae, Tribe Cervini |
| AY748454 | *Cervus elaphus* | Order Artiodactyla, Suborder Ruminantia, Family Cervidae, Tribe Cervini |
| FJ436714 | *Cervus elaphus* | Order Artiodactyla, Suborder Ruminantia, Family Cervidae, Tribe Cervini |
| FJ436716 | *Cervus elaphus* | Order Artiodactyla, Suborder Ruminantia, Family Cervidae, Tribe Cervini |
| FJ436713 | *Cervus elaphus* | Order Artiodactyla, Suborder Ruminantia, Family Cervidae, Tribe Cervini |
| FJ436715 | *Cervus elaphus* | Order Artiodactyla, Suborder Ruminantia, Family Cervidae, Tribe Cervini |
| AF113941 | *Cervus nippon* | Order Artiodactyla, Suborder Ruminantia, Family Cervidae, Tribe Cervini |
| AY286007 | *Dama dama* | Order Artiodactyla, Suborder Ruminantia, Family Cervidae, Tribe Cervini |
| KC476497 | *Elaphurus davidianus* | Order Artiodactyla, Suborder Ruminantia, Family Cervidae, Tribe Cervini |
| MW804583 | *Elaphurus davidianus* | Order Artiodactyla, Suborder Ruminantia, Family Cervidae, Tribe Cervini |
| DQ358970 | *Cervus nippon* | Order Artiodactyla, Suborder Ruminantia, Family Cervidae, Tribe Cervini |
| EF057409 | *Cervus nippon* | Order Artiodactyla, Suborder Ruminantia, Family Cervidae, Tribe Cervini |
| MK103019 | *Cervus nippon* | Order Artiodactyla, Suborder Ruminantia, Family Cervidae, Tribe Cervini |
| OL961483 | *Rucervus eldii* | Order Artiodactyla, Suborder Ruminantia, Family Cervidae, Tribe Cervini |
| OL961484 | *Rucervus eldii* | Order Artiodactyla, Suborder Ruminantia, Family Cervidae, Tribe Cervini |
| OL961485 | *Rucervus eldii* | Order Artiodactyla, Suborder Ruminantia, Family Cervidae, Tribe Cervini |
| MH230114 | *Alces alces* | Order Artiodactyla, Suborder Ruminantia, Family Cervidae, Tribe Odocoileini |
| MH230115 | *Alces alces* | Order Artiodactyla, Suborder Ruminantia, Family Cervidae, Tribe Odocoileini |
| AY639095 | *Alces alces* | Order Artiodactyla, Suborder Ruminantia, Family Cervidae, Tribe Odocoileini |
| JQ290077 | *Alces alces* | Order Artiodactyla, Suborder Ruminantia, Family Cervidae, Tribe Odocoileini |
| MN970212 | *Alces alces* | Order Artiodactyla, Suborder Ruminantia, Family Cervidae, Tribe Odocoileini |
| AY639096 | *Capreolus capreolus* | Order Artiodactyla, Suborder Ruminantia, Family Cervidae, Tribe Odocoileini |
| EF139176 | *Capreolus capreolus* | Order Artiodactyla, Suborder Ruminantia, Family Cervidae, Tribe Odocoileini |
| MK103024 | *Hydropotes inermis* | Order Artiodactyla, Suborder Ruminantia, Family Cervidae, Tribe Odocoileini |
| MK103025 | *Hydropotes inermis* | Order Artiodactyla, Suborder Ruminantia, Family Cervidae, Tribe Odocoileini |
| MK103026 | *Hydropotes inermis* | Order Artiodactyla, Suborder Ruminantia, Family Cervidae, Tribe Odocoileini |
| DQ358969 | *Hydropotes inermis* | Order Artiodactyla, Suborder Ruminantia, Family Cervidae, Tribe Odocoileini |
| EF192236 | *Hydropotes inermis* | Order Artiodactyla, Suborder Ruminantia, Family Cervidae, Tribe Odocoileini |
| EF192237 | *Hydropotes inermis* | Order Artiodactyla, Suborder Ruminantia, Family Cervidae, Tribe Odocoileini |
| U97331 | *Odocoileus hemionus* | Order Artiodactyla, Suborder Ruminantia, Family Cervidae, Tribe Odocoileini |
| AF009180 | *Odocoileus hemionus* | Order Artiodactyla, Suborder Ruminantia, Family Cervidae, Tribe Odocoileini |
| AF009181 | *Odocoileus hemionus* | Order Artiodactyla, Suborder Ruminantia, Family Cervidae, Tribe Odocoileini |
| AY228473 | *Odocoileus hemionus* | Order Artiodactyla, Suborder Ruminantia, Family Cervidae, Tribe Odocoileini |
| AY286008 | *Odocoileus virginianus* | Order Artiodactyla, Suborder Ruminantia, Family Cervidae, Tribe Odocoileini |
| MG856905 | *Odocoileus virginianus* | Order Artiodactyla, Suborder Ruminantia, Family Cervidae, Tribe Odocoileini |
| MG856906 | *Odocoileus virginianus* | Order Artiodactyla, Suborder Ruminantia, Family Cervidae, Tribe Odocoileini |
| MG856907 | *Odocoileus virginianus* | Order Artiodactyla, Suborder Ruminantia, Family Cervidae, Tribe Odocoileini |
| MG856908 | *Odocoileus virginianus* | Order Artiodactyla, Suborder Ruminantia, Family Cervidae, Tribe Odocoileini |
| MG856909 | *Odocoileus virginianus* | Order Artiodactyla, Suborder Ruminantia, Family Cervidae, Tribe Odocoileini |
| MG856910 | *Odocoileus virginianus* | Order Artiodactyla, Suborder Ruminantia, Family Cervidae, Tribe Odocoileini |
| MG856911 | *Odocoileus virginianus* | Order Artiodactyla, Suborder Ruminantia, Family Cervidae, Tribe Odocoileini |
| MG856912 | *Odocoileus virginianus* | Order Artiodactyla, Suborder Ruminantia, Family Cervidae, Tribe Odocoileini |
| MG856913 | *Odocoileus virginianus* | Order Artiodactyla, Suborder Ruminantia, Family Cervidae, Tribe Odocoileini |
| MG856914 | *Odocoileus virginianus* | Order Artiodactyla, Suborder Ruminantia, Family Cervidae, Tribe Odocoileini |
| MG856915 | *Odocoileus virginianus* | Order Artiodactyla, Suborder Ruminantia, Family Cervidae, Tribe Odocoileini |
| MG856916 | *Odocoileus virginianus* | Order Artiodactyla, Suborder Ruminantia, Family Cervidae, Tribe Odocoileini |
| MG856917 | *Odocoileus virginianus* | Order Artiodactyla, Suborder Ruminantia, Family Cervidae, Tribe Odocoileini |
| MG856918 | *Odocoileus virginianus* | Order Artiodactyla, Suborder Ruminantia, Family Cervidae, Tribe Odocoileini |
| MG856919 | *Odocoileus virginianus* | Order Artiodactyla, Suborder Ruminantia, Family Cervidae, Tribe Odocoileini |
| MG856920 | *Odocoileus virginianus* | Order Artiodactyla, Suborder Ruminantia, Family Cervidae, Tribe Odocoileini |
| MG856921 | *Odocoileus virginianus* | Order Artiodactyla, Suborder Ruminantia, Family Cervidae, Tribe Odocoileini |
| MG856922 | *Odocoileus virginianus* | Order Artiodactyla, Suborder Ruminantia, Family Cervidae, Tribe Odocoileini |
| MG856923 | *Odocoileus virginianus* | Order Artiodactyla, Suborder Ruminantia, Family Cervidae, Tribe Odocoileini |
| MG856924 | *Odocoileus virginianus* | Order Artiodactyla, Suborder Ruminantia, Family Cervidae, Tribe Odocoileini |
| MG856925 | *Odocoileus virginianus* | Order Artiodactyla, Suborder Ruminantia, Family Cervidae, Tribe Odocoileini |
| MG856926 | *Odocoileus virginianus* | Order Artiodactyla, Suborder Ruminantia, Family Cervidae, Tribe Odocoileini |
| MG856927 | *Odocoileus virginianus* | Order Artiodactyla, Suborder Ruminantia, Family Cervidae, Tribe Odocoileini |
| MG856928 | *Odocoileus virginianus* | Order Artiodactyla, Suborder Ruminantia, Family Cervidae, Tribe Odocoileini |
| MG856929 | *Odocoileus virginianus* | Order Artiodactyla, Suborder Ruminantia, Family Cervidae, Tribe Odocoileini |
| MG856930 | *Odocoileus virginianus* | Order Artiodactyla, Suborder Ruminantia, Family Cervidae, Tribe Odocoileini |
| MZ773901 | *Odocoileus virginianus* | Order Artiodactyla, Suborder Ruminantia, Family Cervidae, Tribe Odocoileini |
| XM020883647 | *Odocoileus virginianus* | Order Artiodactyla, Suborder Ruminantia, Family Cervidae, Tribe Odocoileini |
| EU032303 | *Rangifer tarandus* | Order Artiodactyla, Suborder Ruminantia, Family Cervidae, Tribe Odocoileini |
| MN052815 | *Rangifer tarandus* | Order Artiodactyla, Suborder Ruminantia, Family Cervidae, Tribe Odocoileini |
| MN052816 | *Rangifer tarandus* | Order Artiodactyla, Suborder Ruminantia, Family Cervidae, Tribe Odocoileini |
| MN052817 | *Rangifer tarandus* | Order Artiodactyla, Suborder Ruminantia, Family Cervidae, Tribe Odocoileini |
| MN052818 | *Rangifer tarandus* | Order Artiodactyla, Suborder Ruminantia, Family Cervidae, Tribe Odocoileini |
| MN052819 | *Rangifer tarandus* | Order Artiodactyla, Suborder Ruminantia, Family Cervidae, Tribe Odocoileini |
| MN052820 | *Rangifer tarandus* | Order Artiodactyla, Suborder Ruminantia, Family Cervidae, Tribe Odocoileini |
| MT361767 | *Rangifer tarandus* | Order Artiodactyla, Suborder Ruminantia, Family Cervidae, Tribe Odocoileini |
| MT361766 | *Rangifer tarandus* | Order Artiodactyla, Suborder Ruminantia, Family Cervidae, Tribe Odocoileini |
| AY639093 | *Rangifer tarandus* | Order Artiodactyla, Suborder Ruminantia, Family Cervidae, Tribe Odocoileini |
| MK097270 | *Rangifer tarandus* | Order Artiodactyla, Suborder Ruminantia, Family Cervidae, Tribe Odocoileini |
| MN784958 | *Rangifer tarandus* | Order Artiodactyla, Suborder Ruminantia, Family Cervidae, Tribe Odocoileini |
| MN784959 | *Rangifer tarandus* | Order Artiodactyla, Suborder Ruminantia, Family Cervidae, Tribe Odocoileini |
| MN784960 | *Rangifer tarandus* | Order Artiodactyla, Suborder Ruminantia, Family Cervidae, Tribe Odocoileini |
| MN784961 | *Rangifer tarandus* | Order Artiodactyla, Suborder Ruminantia, Family Cervidae, Tribe Odocoileini |
| MW557843 | *Rangifer tarandus* | Order Artiodactyla, Suborder Ruminantia, Family Cervidae, Tribe Odocoileini |
| MW557844 | *Rangifer tarandus* | Order Artiodactyla, Suborder Ruminantia, Family Cervidae, Tribe Odocoileini |
| MW557845 | *Rangifer tarandus* | Order Artiodactyla, Suborder Ruminantia, Family Cervidae, Tribe Odocoileini |
| AF113944 | *Muntiacus muntjak* | Order Artiodactyla, Suborder Ruminantia, Family Cervidae, Tribe Muntiacini |
| KC788406 | *Muntiacus reevesi* | Order Artiodactyla, Suborder Ruminantia, Family Cervidae, Tribe Muntiacini |
| AB809458 | *Muntiacus reevesi* | Order Artiodactyla, Suborder Ruminantia, Family Cervidae, Tribe Muntiacini |
| MK103021 | *Muntiacus reevesi* | Order Artiodactyla, Suborder Ruminantia, Family Cervidae, Tribe Muntiacini |
| MK103023 | *Muntiacus reevesi* | Order Artiodactyla, Suborder Ruminantia, Family Cervidae, Tribe Muntiacini |
| EF165086 | *Connochaetes taurinus* | Order Artiodactyla, Suborder Ruminantia, Family Bovidae, Tribe Alcelaphini |
| AY720705 | *Antilope cervicapra* | Order Artiodactyla, Suborder Ruminantia, Family Bovidae, Tribe Antilopini |
| AF117313 | *Gazella subgutturosa* | Order Artiodactyla, Suborder Ruminantia, Family Bovidae, Tribe Antilopini |
| EU032301 | *Gazella thomsonii* | Order Artiodactyla, Suborder Ruminantia, Family Bovidae, Tribe Antilopini |
| AB473602 | *Procapra gutturosa* | Order Artiodactyla, Suborder Ruminantia, Family Bovidae, Tribe Antilopini |
| AB473603 | *Procapra gutturosa* | Order Artiodactyla, Suborder Ruminantia, Family Bovidae, Tribe Antilopini |
| AB473604 | *Procapra gutturosa* | Order Artiodactyla, Suborder Ruminantia, Family Bovidae, Tribe Antilopini |
| AB473605 | *Procapra gutturosa* | Order Artiodactyla, Suborder Ruminantia, Family Bovidae, Tribe Antilopini |
| AB473606 | *Procapra gutturosa* | Order Artiodactyla, Suborder Ruminantia, Family Bovidae, Tribe Antilopini |
| AB473607 | *Procapra gutturosa* | Order Artiodactyla, Suborder Ruminantia, Family Bovidae, Tribe Antilopini |
| AB473608 | *Procapra gutturosa* | Order Artiodactyla, Suborder Ruminantia, Family Bovidae, Tribe Antilopini |
| AB473610 | *Procapra gutturosa* | Order Artiodactyla, Suborder Ruminantia, Family Bovidae, Tribe Antilopini |
| AB473612 | *Procapra gutturosa* | Order Artiodactyla, Suborder Ruminantia, Family Bovidae, Tribe Antilopini |
| AB473613 | *Procapra gutturosa* | Order Artiodactyla, Suborder Ruminantia, Family Bovidae, Tribe Antilopini |
| AB473615 | *Procapra gutturosa* | Order Artiodactyla, Suborder Ruminantia, Family Bovidae, Tribe Antilopini |
| AY720695 | *Bison bison* | Order Artiodactyla, Suborder Ruminantia, Family Bovidae, Tribe Bovini |
| AF117328 | *Bison bonasus* | Order Artiodactyla, Suborder Ruminantia, Family Bovidae, Tribe Bovini |
| EU032297 | *Bison bonasus* | Order Artiodactyla, Suborder Ruminantia, Family Bovidae, Tribe Bovini |
| EU032298 | *Bison bonasus* | Order Artiodactyla, Suborder Ruminantia, Family Bovidae, Tribe Bovini |
| EU032300 | *Bos frontalis* | Order Artiodactyla, Suborder Ruminantia, Family Bovidae, Tribe Bovini |
| AB534900 | *Bos frontalis* | Order Artiodactyla, Suborder Ruminantia, Family Bovidae, Tribe Bovini |
| AB534901 | *Bos frontalis* | Order Artiodactyla, Suborder Ruminantia, Family Bovidae, Tribe Bovini |
| AY720697 | *Bos gaurus* | Order Artiodactyla, Suborder Ruminantia, Family Bovidae, Tribe Bovini |
| AY367635 | *Bos grunniens* | Order Artiodactyla, Suborder Ruminantia, Family Bovidae, Tribe Bovini |
| AY327450 | *Bos grunniens* | Order Artiodactyla, Suborder Ruminantia, Family Bovidae, Tribe Bovini |
| KC137635 | *Bos grunniens* | Order Artiodactyla, Suborder Ruminantia, Family Bovidae, Tribe Bovini |
| KC137636 | *Bos grunniens* | Order Artiodactyla, Suborder Ruminantia, Family Bovidae, Tribe Bovini |
| KC137637 | *Bos grunniens* | Order Artiodactyla, Suborder Ruminantia, Family Bovidae, Tribe Bovini |
| KC137638 | *Bos grunniens* | Order Artiodactyla, Suborder Ruminantia, Family Bovidae, Tribe Bovini |
| KC137639 | *Bos grunniens* | Order Artiodactyla, Suborder Ruminantia, Family Bovidae, Tribe Bovini |
| KC137643 | *Bos grunniens* | Order Artiodactyla, Suborder Ruminantia, Family Bovidae, Tribe Bovini |
| KC137645 | *Bos grunniens* | Order Artiodactyla, Suborder Ruminantia, Family Bovidae, Tribe Bovini |
| KC137646 | *Bos grunniens* | Order Artiodactyla, Suborder Ruminantia, Family Bovidae, Tribe Bovini |
| AY720515 | *Bos indicus* | Order Artiodactyla, Suborder Ruminantia, Family Bovidae, Tribe Bovini |
| EU564453 | *Bos indicus* | Order Artiodactyla, Suborder Ruminantia, Family Bovidae, Tribe Bovini |
| AB534904 | *Bos indicus* | Order Artiodactyla, Suborder Ruminantia, Family Bovidae, Tribe Bovini |
| AB534905 | *Bos indicus* | Order Artiodactyla, Suborder Ruminantia, Family Bovidae, Tribe Bovini |
| AY720691 | *Bos javanicus* | Order Artiodactyla, Suborder Ruminantia, Family Bovidae, Tribe Bovini |
| AY720692 | *Bos javanicus* | Order Artiodactyla, Suborder Ruminantia, Family Bovidae, Tribe Bovini |
| AY720693 | *Bos javanicus* | Order Artiodactyla, Suborder Ruminantia, Family Bovidae, Tribe Bovini |
| D10613 | *Bos primigenius* | Order Artiodactyla, Suborder Ruminantia, Family Bovidae, Tribe Bovini |
| D10614 | *Bos primigenius* | Order Artiodactyla, Suborder Ruminantia, Family Bovidae, Tribe Bovini |
| D10612 | *Bos taurus* | Order Artiodactyla, Suborder Ruminantia, Family Bovidae, Tribe Bovini |
| AF455119 | *Bos taurus* | Order Artiodactyla, Suborder Ruminantia, Family Bovidae, Tribe Bovini |
| AY247262 | *Bos taurus* | Order Artiodactyla, Suborder Ruminantia, Family Bovidae, Tribe Bovini |
| AY367639 | *Bos taurus* | Order Artiodactyla, Suborder Ruminantia, Family Bovidae, Tribe Bovini |
| DQ112003 | *Bos taurus* | Order Artiodactyla, Suborder Ruminantia, Family Bovidae, Tribe Bovini |
| DQ333350 | *Bos taurus* | Order Artiodactyla, Suborder Ruminantia, Family Bovidae, Tribe Bovini |
| EF626475 | *Bos taurus* | Order Artiodactyla, Suborder Ruminantia, Family Bovidae, Tribe Bovini |
| EU224467 | *Bos taurus* | Order Artiodactyla, Suborder Ruminantia, Family Bovidae, Tribe Bovini |
| AY720699 | *Boselaphus tragocamelus* | Order Artiodactyla, Suborder Ruminantia, Family Bovidae, Tribe Bovini |
| AY720700 | *Boselaphus tragocamelus* | Order Artiodactyla, Suborder Ruminantia, Family Bovidae, Tribe Bovini |
| AY320365 | *Bubalus bubalis* | Order Artiodactyla, Suborder Ruminantia, Family Bovidae, Tribe Bovini |
| AY720689 | *Bubalus bubalis* | Order Artiodactyla, Suborder Ruminantia, Family Bovidae, Tribe Bovini |
| AY768533 | *Bubalus bubalis* | Order Artiodactyla, Suborder Ruminantia, Family Bovidae, Tribe Bovini |
| AY768534 | *Bubalus bubalis* | Order Artiodactyla, Suborder Ruminantia, Family Bovidae, Tribe Bovini |
| AY769894 | *Bubalus bubalis* | Order Artiodactyla, Suborder Ruminantia, Family Bovidae, Tribe Bovini |
| KC137622 | *Bubalus bubalis* | Order Artiodactyla, Suborder Ruminantia, Family Bovidae, Tribe Bovini |
| KC137623 | *Bubalus bubalis* | Order Artiodactyla, Suborder Ruminantia, Family Bovidae, Tribe Bovini |
| KC137625 | *Bubalus bubalis* | Order Artiodactyla, Suborder Ruminantia, Family Bovidae, Tribe Bovini |
| KC137628 | *Bubalus bubalis* | Order Artiodactyla, Suborder Ruminantia, Family Bovidae, Tribe Bovini |
| KC137629 | *Bubalus bubalis* | Order Artiodactyla, Suborder Ruminantia, Family Bovidae, Tribe Bovini |
| KC137630 | *Bubalus bubalis* | Order Artiodactyla, Suborder Ruminantia, Family Bovidae, Tribe Bovini |
| KC137631 | *Bubalus bubalis* | Order Artiodactyla, Suborder Ruminantia, Family Bovidae, Tribe Bovini |
| KC137632 | *Bubalus bubalis* | Order Artiodactyla, Suborder Ruminantia, Family Bovidae, Tribe Bovini |
| KC137633 | *Bubalus bubalis* | Order Artiodactyla, Suborder Ruminantia, Family Bovidae, Tribe Bovini |
| MK342630 | *Bubalus bubalis* | Order Artiodactyla, Suborder Ruminantia, Family Bovidae, Tribe Bovini |
| AY720687 | *Bubalus depressicornis* | Order Artiodactyla, Suborder Ruminantia, Family Bovidae, Tribe Bovini |
| AY720688 | *Bubalus depressicornis* | Order Artiodactyla, Suborder Ruminantia, Family Bovidae, Tribe Bovini |
| AY720685 | *Syncerus caffer* | Order Artiodactyla, Suborder Ruminantia, Family Bovidae, Tribe Bovini |
| AY720686 | *Syncerus caffer* | Order Artiodactyla, Suborder Ruminantia, Family Bovidae, Tribe Bovini |
| AY720683 | *Syncerus caffer* | Order Artiodactyla, Suborder Ruminantia, Family Bovidae, Tribe Bovini |
| AY720684 | *Syncerus caffer* | Order Artiodactyla, Suborder Ruminantia, Family Bovidae, Tribe Bovini |
| MZ508002 | *Ammotragus lervia* | Order Artiodactyla, Suborder Ruminantia, Family Bovidae, Tribe Caprini |
| MG214331 | *Arabitragus jayakari* | Order Artiodactyla, Suborder Ruminantia, Family Bovidae, Tribe Caprini |
| AF117326 | *Budorcas taxicolor* | Order Artiodactyla, Suborder Ruminantia, Family Bovidae, Tribe Caprini |
| AB060290 | *Budorcas taxicolor* | Order Artiodactyla, Suborder Ruminantia, Family Bovidae, Tribe Caprini |
| MG214330 | *Capra falconeri* | Order Artiodactyla, Suborder Ruminantia, Family Bovidae, Tribe Caprini |
| X74758 | *Capra hircus* | Order Artiodactyla, Suborder Ruminantia, Family Bovidae, Tribe Caprini |
| X91999 | *Capra hircus* | Order Artiodactyla, Suborder Ruminantia, Family Bovidae, Tribe Caprini |
| AF486134 | *Capra hircus* | Order Artiodactyla, Suborder Ruminantia, Family Bovidae, Tribe Caprini |
| AF486135 | *Capra hircus* | Order Artiodactyla, Suborder Ruminantia, Family Bovidae, Tribe Caprini |
| AF486136 | *Capra hircus* | Order Artiodactyla, Suborder Ruminantia, Family Bovidae, Tribe Caprini |
| AF486137 | *Capra hircus* | Order Artiodactyla, Suborder Ruminantia, Family Bovidae, Tribe Caprini |
| AF486138 | *Capra hircus* | Order Artiodactyla, Suborder Ruminantia, Family Bovidae, Tribe Caprini |
| DQ013244 | *Capra hircus* | Order Artiodactyla, Suborder Ruminantia, Family Bovidae, Tribe Caprini |
| DQ345066 | *Capra hircus* | Order Artiodactyla, Suborder Ruminantia, Family Bovidae, Tribe Caprini |
| DQ345067 | *Capra hircus* | Order Artiodactyla, Suborder Ruminantia, Family Bovidae, Tribe Caprini |
| DQ345068 | *Capra hircus* | Order Artiodactyla, Suborder Ruminantia, Family Bovidae, Tribe Caprini |
| AF117319 | *Capra ibex* | Order Artiodactyla, Suborder Ruminantia, Family Bovidae, Tribe Caprini |
| MG214329 | *Naemorhedus griseus* | Order Artiodactyla, Suborder Ruminantia, Family Bovidae, Tribe Caprini |
| MW194318 | *Pantholops hodgsonii* | Order Artiodactyla, Suborder Ruminantia, Family Bovidae, Tribe Caprini |
| EF999825 | *Oreamnos americanus* | Order Artiodactyla, Suborder Ruminantia, Family Bovidae, Tribe Caprini |
| XM040247920 | *Oryx dammah* | Order Artiodactyla, Suborder Ruminantia, Family Bovidae, Tribe Caprini |
| DQ149351 | *Ovis aries* | Order Artiodactyla, Suborder Ruminantia, Family Bovidae, Tribe Caprini |
| AY907685 | *Ovis aries* | Order Artiodactyla, Suborder Ruminantia, Family Bovidae, Tribe Caprini |
| DQ149332 | *Ovis aries* | Order Artiodactyla, Suborder Ruminantia, Family Bovidae, Tribe Caprini |
| DQ272610 | *Ovis aries* | Order Artiodactyla, Suborder Ruminantia, Family Bovidae, Tribe Caprini |
| DQ149333 | *Ovis aries* | Order Artiodactyla, Suborder Ruminantia, Family Bovidae, Tribe Caprini |
| AF166334 | *Ovis canadensis* | Order Artiodactyla, Suborder Ruminantia, Family Bovidae, Tribe Caprini |
| AJ969015 | *Ovis dalli* | Order Artiodactyla, Suborder Ruminantia, Family Bovidae, Tribe Caprini |
| AJ969016 | *Ovis dalli* | Order Artiodactyla, Suborder Ruminantia, Family Bovidae, Tribe Caprini |
| MG214327 | *Ovis vignei* | Order Artiodactyla, Suborder Ruminantia, Family Bovidae, Tribe Caprini |
| MG214328 | *Ovis vignei* | Order Artiodactyla, Suborder Ruminantia, Family Bovidae, Tribe Caprini |
| KT845867 | *Rupicapra pyrenaica* | Order Artiodactyla, Suborder Ruminantia, Family Bovidae, Tribe Caprini |
| KT845868 | *Rupicapra pyrenaica* | Order Artiodactyla, Suborder Ruminantia, Family Bovidae, Tribe Caprini |
| AY735496 | *Rupicapra rupicapra* | Order Artiodactyla, Suborder Ruminantia, Family Bovidae, Tribe Caprini |
| EF139173 | *Rupicapra rupicapra* | Order Artiodactyla, Suborder Ruminantia, Family Bovidae, Tribe Caprini |
| AF117309 | *Addax nasomaculatus* | Order Artiodactyla, Suborder Ruminantia, Family Bovidae, Tribe Hippotragini |
| AF117323 | *Hippotragus niger* | Order Artiodactyla, Suborder Ruminantia, Family Bovidae, Tribe Hippotragini |
| EF165084 | *Hippotragus niger* | Order Artiodactyla, Suborder Ruminantia, Family Bovidae, Tribe Hippotragini |
| EF165085 | *Hippotragus niger* | Order Artiodactyla, Suborder Ruminantia, Family Bovidae, Tribe Hippotragini |
| EF165087 | *Kobus ellipsiprymnus* | Order Artiodactyla, Suborder Ruminantia, Family Bovidae, Tribe Reduncini |
| EF165088 | *Kobus megaceros* | Order Artiodactyla, Suborder Ruminantia, Family Bovidae, Tribe Reduncini |
| EU032302 | *Kobus ellipsiprymnus* | Order Artiodactyla, Suborder Ruminantia, Family Bovidae, Tribe Reduncini |
| EF165082 | *Tragelaphus oryx* | Order Artiodactyla, Suborder Ruminantia, Family Bovidae, Tribe Tragelaphini |
| X74771 | *Tragelaphus strepsiceros* | Order Artiodactyla, Suborder Ruminantia, Family Bovidae, Tribe Tragelaphini |
| X74759 | *Tragelaphus strepsiceros* | Order Artiodactyla, Suborder Ruminantia, Family Bovidae, Tribe Tragelaphini |
| AY720701 | *Tragelaphus strepsiceros* | Order Artiodactyla, Suborder Ruminantia, Family Bovidae, Tribe Tragelaphini |
| EF165081 | *Tragelaphus strepsiceros* | Order Artiodactyla, Suborder Ruminantia, Family Bovidae, Tribe Tragelaphini |
| AY720703 | *Tragelaphus imberbis* | Order Artiodactyla, Suborder Ruminantia, Family Bovidae, Tribe Tragelaphini |
| AF117321 | *Tragelaphus angasi* | Order Artiodactyla, Suborder Ruminantia, Family Bovidae, Tribe Tragelaphini |
| EU032295 | *Tragelaphus angasii* | Order Artiodactyla, Suborder Ruminantia, Family Bovidae, Tribe Tragelaphini |
| EU032296 | *Tragelaphus angasii* | Order Artiodactyla, Suborder Ruminantia, Family Bovidae, Tribe Tragelaphini |
| EF165083 | *Tragelaphus spekii* | Order Artiodactyla, Suborder Ruminantia, Family Bovidae, Tribe Tragelaphini |
